# Is Basal Cell Carcinoma an Itchy Tumor? Clinical Characteristics of Itch in Basal Cell Carcinoma

**DOI:** 10.3390/jcm9082386

**Published:** 2020-07-26

**Authors:** Iwona Chlebicka, Aleksandra A Stefaniak, Łukasz Matusiak, Jacek C Szepietowski

**Affiliations:** Department of Dermatology, Venereology and Allergology, Wroclaw Medical University, Poland, ul. Chałubińskiego 1, 50-368 Wrocław, Poland; iwonak4wsk@interia.pl (I.C.); aleksandraannastefaniak@gmail.com (A.A.S.); luke71@interia.pl (L.M.)

**Keywords:** basal cell carcinoma, itch, pruritus

## Abstract

In common knowledge, basal cell carcinoma (BCC) is known to be asymptomatic, but in clinical practice, at least some patients complain of itching. The present study group comprised of 180 patients with histologically confirmed BCC. Detailed information on demographics, clinical history, and physical findings was recorded. Moreover, various clinical features of itch (including intensity, localization, quality, descriptors) and the most common factors responsible for its aggravation or alleviation were examined. The itch was present in 21.1% of patients with BCC and was limited to the tumor. The mean intensity of the itch was 3.1 ± 1.2 points (Numerical Rating Scale), indicating moderate itch intensity. Among the patients, 22.4% reported that itching occurred constantly, every day. BCC-associated itch seems to be moderately frequent, although being a seemingly underestimated problem among patients with BCC.

## 1. Introduction

Basal cell carcinoma (BCC) is the most common skin cancer, accounting for 80% of all cases, followed by squamous cell carcinoma (SCC) (16%) and melanoma (4%) [1]. Despite the frequency of BCC, BCC-associated data on frequently encountered dermatologic symptoms are still emerging. In common knowledge, most skin tumors are known to be asymptomatic, but in clinical practice, at least some patients complain of itching. However, only a few studies have investigated itch in skin tumors [2,3]. There is also very limited knowledge on the intensity of itching which could accompany BCCs [4,5].

Therefore, the aim of this study was to assess the prevalence and clinical characteristics of itch in BCC patients.

## 2. Material and Methods

### 2.1. Patients

The material for the study was collected between September 2017 and December 2019. The project was approved by the local Ethical Committee (ST.C260.18.019). The study procedures were carried out in agreement with the Helsinki Declaration of 1964 and its later amendments, and good clinical practice guidelines. Written informed consent to participate was obtained from all patients before enrollment, and they were also informed of their right to leave the study at any time. We approached 211 consecutive patients above the age of 17 who were surgically treated in the Dermatosurgery Unit with the clinical diagnosis of BCC. Twenty patients refused participation in the study without specifying the reasons (response rate: 90.5%). The inclusion criterion was BCC as the initial primary clinical diagnosis. Exclusion criteria included: mental status changes making the patient unable to make a detailed assessment of itch, known severe renal or liver disease, or a history of chronic dermatological or psychiatric diseases. After the surgery, the lesions were verified with histopathology. Among 191 patients, 11 subjects’ histopathology revealed lesions other than BCC. Those patients were excluded from the study. Therefore, the final study group constituted 180 patients (83 women and 97 men) with histologically confirmed BCC. The age range of studied individuals was 17–95 years (mean: 70.5 ± 11.9 years). All patients in our study group were Caucasians. Individuals with itch constituted a group for further detailed analysis.

### 2.2. Study Design and Itch Assessment

After inclusion, detailed information on demographics, clinical history, and physical findings was recorded. All BCCs were divided into the following clinical types: nodular, superficial, morphoeic, and ulcerated [6].

The main clinical parameter, the presence of itching, was documented, including the affected anatomical locations. A numerical rating scale (NRS; 0: no itch; 10 points: worst imaginable itch) was utilized to assess maximal values of itch intensity both in the last 3 days and during the course of the tumor. NRS cut-off points were used as follows: 1–<3 points represent mild itch; 3–6 points, moderate itch; ≥7–8 points, severe itch; and ≥9 points, very severe itch [7]. Additionally, the Four-Item Itch Questionnaire (4IIQ), previously used by our group in many studies on different types of itch [8,9,10], was employed to estimate the extent (1–3 points), intensity (1–5 points), frequency (1–5 points), and sleep disturbances (0–6 points) caused by itching. In this scoring, ratings range from 3 (mild pruritus) to 19 points (very severe pruritus). Furthermore, various clinical features of itching were assessed, including its exact localization, quality, and description of cutaneous sensations associated with the itch. Moreover, certain factors influencing itch intensity were also examined. The clinical characteristics of itch will be presented in two ways—quantitatively, by assessing the intensity of itch in the NRS and 4IIQ scales, as well as descriptively.

### 2.3. Statistical Analysis

All variables were assessed for normal or non-normal distribution, in order to apply corresponding statistical tests. Differences between groups were determined using the Mann–Whitney U test with reference to the non-normal distribution of evaluated continuous variables. Moreover, the comparisons between groups were conducted using Pearson’s Chi-squared test for categorical sets of variables. Correlations were determined using Spearman’s correlation analysis. The level of significance was set at α = 0.05. The resulting p-values were considered significant if *p* < 0.05. Statistical analyses were performed using Statistica 12 software (StatSoft, Tulsa, OK, USA). The prevalence of itch in BCC patients will be expressed as a percentage.

## 3. Results

Itch was present in 31.1% (56/180) of patients with BCC. Nearly half of the patients (48.2%) experienced itching a few times per week, while the remaining subjects experienced itching every day (21.4%) or at least a few times per month (10.7%). Among the patients, 12.5% experienced itching fewer than once a month. Out of 56 itchy subjects, 41 patients (71.4%) had itching present during the last 3 days. According to NRS, the maximum intensity of itching during the whole period of existing tumors was assessed as 3.4 ± 1.8 points (range: 1–8 points; median: 3 points) and during last 3 days as 3.1 ± 1.2 points (range: 1–5 points; median: 3 points). Mild itching during the last 3 days was observed in 27.5% of patients, and moderate itching in the remaining 72.5% of patients. Severe or very severe itching was not documented. The mean result for 4IIQ was estimated at 4.3 ± 2.4 points (range: 3–15 points) and significantly correlated with NRS during the last 3 days and during the whole period of the existing tumor (r = 0.36, *p* = 0.01 and r = 0.32 *p* = 0.02, respectively).

Nearly one-third of the subjects (32.7%) responded that they had experienced maximal itch intensity while the tumor was already fully developed—23.6% at the beginning of the tumor’s growth, and 9.1% when the tumor was expanding. Of the itchy subjects, 34.6% did not find any relationship in accordance with tumor growth. Usually, itch was not accompanied by other unpleasant cutaneous sensations (48.2%). Less commonly, it was described as stinging (10.7%), burning (8.9%), or tingling (7.1%). Patients frequently described the itch as annoying (43.9%), distressing (34.1%), and burdensome (34.1%).

Itch in all patients with BCC was limited to the tumor. There was not even a single subject reporting generalized itch. Itch requiring scratching was found in 57.9% of individuals and appeared mostly as short itch episodes with a duration less than 1 min (67.2%). Most of the patients (75.8%) could not identify whether the itch tended to appear more frequently at any specific time of day (morning, afternoon, evening, or night).

Sleep disturbances, described as “awakenings during the night’s sleep due to itching”, were observed in 12.1% of patients, and two patients (3.6%) reported the use of soporifics. The most common exacerbating factors included sweat (13.8%), and equally, stress, fatigue, and physical activity (12.3% each), while most of the patients (86.8%) did not see any correlation between analyzed factors and itch alleviation.

There were no relationships between itch presence and clinical type of the tumor.

Moreover, no significant differences in gender, age, body mass index (BMI), duration of the disease, clinical type, diameter, clinical presence of the ulceration, or number of tumors were found between the patients with itch and those without itch (*p* > 0.05) (Table 1).

Regarding the treatment for itch, subjects were most frequently using nothing at all (71%) to alleviate itching, while 16.1% were using non-specific ointment (emollient) and 12.9% of subjects used self-massage. There was not even a single subject who received specific antipruritic treatment from their doctor.

## 4. Discussion

The prevalence of itching in our patients with BCC (31.1%) remains in concordance with previous reports in which this symptom was diagnosed in 14.5–33.4% of subjects [2,11,12,13] (Table 2). The itch prevalence in BCC seems to be comparable with another non-melanoma skin cancer – SCC (27.3–43%) [3,11,12,13]. Interestingly, itch in melanoma was less common (5.3–28.8%) [3,11,14,15,16]. Compared with hematologic neoplasms, commonly associated with itch, such as polycythemia vera (31-68%) [17,18,19] or Hodgkin’s lymphoma (25–36.2%) [20,21], itching in BCC is less common. However, it occurs more frequently in comparison with non-Hodgkin’s lymphoma (3.2–15.6%) [22,23] or itch in solid tumors (<1%) [24]. In opposition to common skin diseases, such as atopic dermatitis (91–100%) [25,26] or psoriasis (84–95%) [25,27,28], the frequency of itch in patients with BCC is much lower. However, most of all, considering the older age of our studied population (70.5 ± 11.9 years), we may also compare it to the prevalence of itch in elderly people, which we found to be as high as 35.3% in hospitalized subjects in the geriatric ward [29].

Concerning the intensity of itch, in our cohort of patients, itch intensity measured with the NRS score was 3.1 ± 1.2 points, indicating moderate itch intensity. This finding is in accordance with previous reports. Poulaliou et al. [13], in the study done on the French population, reported that the mean intensity of itching in the 5D itch scale score was 9.1 points (range: 5–12 points). In the NRS score, it was 2.7 ± 1.2 points. Yosipovitch et al. [11] in 2012, in his study among 166 BCCs, reported the mean itch intensity of 3.8 ± 0.26 points in the Visual Analogue Scale (VAS); however, they did not exclude from their final analysis patients with preexisting itch disorders.

The mean BCC-associated itch intensity evaluated with NRS remains comparable with the intensity of itch in SCC (3.1–4.0 points) [11,13] and with melanoma-associated itch (3.3 ± 1.03 points) [11]. This intensity may also be commensurate with itch observed in neoplasms, such as polycythemia vera (4.8 ± 1.9 points) [17] or internal diseases, such as uraemic itch (4.1 ± 2.0 points) [30] or acute heart failure (4.2 ± 2.9 points) [31]. However, with regard to itching observed in other frequent dermatologic disorders, such as atopic dermatitis (7.9 ± 2.2 points) [32], psoriasis (6.6 ± 2.6 points) [25], or lichen planus (6.5 ± 2.7 points) [33] the intensity of itch measured with NRS or VAS in patients with BCC is much lower. However, intensity of itch in our cohort of BCC patients was only slightly lower than that reported in elderly patients (3.7 ± 3.0 points) [29]. 

In our cohort of patients, itch was described as stinging (10.7%), burning (8.9%), or tingling (7.1%). Askari et al. [3] compared the presenting signs of melanoma, BCC, SCC, and seborrhoeic keratosis in an elderly male population. Burning was more commonly reported by patients with BCC (4.4%) and SCC (4.2%) than by those with melanoma (0%) and seborrhoeic keratosis (1.8%). Other sensations than burning, according to our best knowledge, were examined only in the French study [13], reporting the prevalence of stinging (13%), tickling (13%), burning (3%), pinching (2%), and biting (0%). There was no significant difference between BCC and SCC, besides the stinging sensations, which were most frequent in SCC but only of borderline significance (*p* = 0.05).

Pathogenesis of itching in BCC is not fully understood, and various factors have been suggested as contributing to the development of this symptom. Currently, it is believed that malignancy-associated itching may be the result of a neoplasm’s local effect, like tissue reaction to toxic products generated by the tumor itself, or due to the systemic reaction, such as allergic reactions to compounds released to malignancy [4]. Yosipovitch et al. [11] found no association between the presence of ulceration and itch, which is in accordance with our findings. In the analysis, it was also revealed an association between the degree of inflammation and itch intensity (*p* = 0.01), similarly to findings presented by Lee et al. [2]. Additionally, the presence of eosinophils was significantly associated with an increased prevalence of itching (*p* = 0.02) [11].

This study has several limitations. Firstly, this was a single center study, and the number of subjects with itching was relatively small. In addition, the descriptive nature of the study has to be taken into account. Secondly, this study population was a relatively homogenous elderly group, which makes it more difficult to generalize to other patient populations. Thirdly, as most patients in our study were of more advanced age, it cannot be excluded that the prevalence of itch related to BCC may be influenced by general itching of the elderly population. Last, but by no means least, concerning the background of itching, we did not consider comorbidities and medications beside severe renal or liver disease, a history of chronic dermatological or psychiatric diseases.

## 5. Conclusions

In conclusion, BCC-associated itch is a moderately frequent, although seemingly underestimated problem among patients with BCC. It should be highlighted that more than 20% of patients reported that itching occurs constantly, every day. The findings of the current study have provided additional information regarding the characteristics of itch in BCC, especially in terms of the sensory and affective components of the itching sensation. However, further multicenter studies, including a reasonable number of patients with BCC, are necessary to confirm our findings.

## Figures and Tables

**Table 1:** Basic demographics and tumor-related data for itchy and non-itchy patients.

	Patients with Itch	Patients without Itch	*p*-Value
**Gender** (*n*)	56	124	NS
Women	27 (48.2%)	56 (45.2%)	
Men	29 (51.8%)	68 (54.8%)	
**Age** (years), Mean ± SD	73.3 ± 11.1	69.4 ± 12	NS
Range	(51–94)	(17–93)	
**BMI** (kg/m^2^), Mean ± SD	27.4 ± 4.3	27.7 ± 4.6	NS
Range	(19.8–40.4)	(19.1–42.1)	
**Duration of Tumor Development** (Months), Mean ± SD	33.2 ± 70.3	20.8 ± 34.4	NS
Range	(0–432)	(0–180)	
**Number of Lesions Per Person** (*n*)	1.1 ± 0.4	1.1 ± 0.3	NS
Range	(1–3)	(1–2)	
**Clinical Type of BCC**			NS
Nodular	50 (89.3%)	112 (90.3%)	
Superficial	4 (7.1%)	8 (6.5%)	
Ulcerative	0	1 (0.8%)	
Morphoeic	0	2 (1.6%)	
No data	2 (3.6%)	1 (0.8%)	
**Location of The Tumor**			NS
Face and Neck	35 (62.5%)	97 (78.2%)	
Scalp Area	8 (14.3%)	12 (9.7%)	
Other Body Sites	12 (21.4%)	15 (12.1%)	
No Data	1 (1.8%)	0	
**Diameter of The Tumor (mm)**			NS
0–19 mm	34 (60.7%)	90 (72.6%)	
More Than 20 mm	19 (33.9%)	31 (25%)	
No Data	3 (5.4%)	3 (2.4%)	
**Presence of the Ulceration**			NS
Present	20 (35.7%)	60 (48.4%)	
Absent	34 (60.7%)	58 (46.8%)	
No Data	2 (3.6%)	6 (4.8%)	

SD- standard deviation; NS- not significant.BCC- Basal Cell Carcinoma, BMI-Body Mass Index.

**Table 2:** Comparison of basal cell carcinoma-associated itch among series of published patients.

Authors	Participants, *n*	Patients Affected by Itch	NRS/VAS Score (Points ± SD)	Itch Descriptors	Factors Correlating With Itch
Lee at al., 2017 [2]	124	14.5%	No data	No Data	Degree of Inflammation
Poulaliou et al., 2016 [13]	83	22%	2.7 ± 1.2	Stinging (13%), Tickling 13%, Burning (3%), Pinching 2% and Biting 0%	Not Revealed
Yosipovitch et al., 2015 [11]	166	31.9%	3.8 ± 0.26	No Data	Degree of Inflammation, Eosinophilic Infiltration
Mills et al., 2012 [12]	223	33.4%	No Data	No Data	Not Revealed
Askari et al., 2007 [3]	411	22.6%	No Data	Burning (4.4%)	Not Revealed
Current Study	180	31.1%	3.1 ± 1.2	Atinging (10.7%), Burning (8.9%), Tickling (7.1%)	Not Revealed

NRS – Numerical rating scale, SD – standard deviation, VAS – Numerical Visual Rating Scale.
